# A New Ceanothane-Type Triterpenoid Saponin Isolated from *Gouania leptostachya* DC. var. *tonkinensis* Pit. and Its Underlying Anti-Inflammatory Effects

**DOI:** 10.4014/jmb.2301.01040

**Published:** 2023-04-24

**Authors:** Nguyen Thi Hang, Nguyen Thi Bich Thu, Le Ba Vinh, Nguyen Viet Phong, Tran Van On, Ki Yong Lee

**Affiliations:** 1 National Institute of Medicinal Materials, Hanoi 11022, Vietnam; 2 Faculty of Pharmacy, Duy Tan University, Danang 550000, Vietnam; 3 Institute of Marine Biochemistry, Vietnam Academy of Science and Technology, Hanoi 100000, Vietnam; 4 Hanoi University of Pharmacy, Le Thanh Tong, Hanoi 11021, Vietnam; 5 College of Pharmacy, Korea University, Sejong 30019, Republic of Korea

**Keywords:** *Gouania leptostachya*, saponin, anti-inflammatory effect, gouanioside A

## Abstract

Metabolites from medicinal plants continue to hold significant value in the exploration and advancement of novel pharmaceuticals. In the search for plants containing compounds with anti-inflammatory effects, we observed that the ethanol (EtOH) extract obtained from the aerial components of *Gouania leptostachya* DC. var. *tonkinensis* Pit. exhibited substantial suppression of nitric oxide (NO) in vitro. In a phytochemical study on an EtOH extract of *G. leptostachya*, 11 compounds were purified, including one unreported compound namely gouanioside A (1). Their chemical structures were unambiguously determined through the use of various spectroscopic techniques, such as 1 and 2D NMR, IR, and HR-ESI-MS, and by producing derivatives via chemical reactions. The EtOH extract, fractions, and a new compound exerted inflammatory effects by altering NO synthesis in murine RAW264.7 macrophage cells stimulated with lipopolysaccharide. The underlying inflammatory mechanism of the new compound **1** was also explored through various in vitro experiments. The results of this study indicate the potential usefulness of new compound **1** from *G. leptostachya* as a treatment for inflammatory diseases.

## Introduction

The process of inflammation is intricate and linked with several factors, including bacterial infection, toxic compounds, and damaged cells [1]. Chronic inflammation is associated with approximately 40 million deaths annually, according to the World Health Organization (WHO). The inflammation process is also frequently associated with the concurrence of diseases, such as coronavirus 2019 [2], allergies [3], diabetes mellitus [4], gut diseases [3], cardiovascular disease [5], and cancer [6].

Nitric oxide (NO) plays an effector role in inflammatory disease. Cytokines such as interleukin 6 (IL-6), prostaglandin E_2_ (PGE_2_), and tumor necrosis factor alpha (TNF-α) are produced by immune effector cells [6, 7]. Thus, the inhibition of NO production and pro-inflammatory cytokines, such as PGE_2_, IL-6, IL-1β, and TNF-α, in addition to the manifestation of proteins such as cyclooxygenase 2 (COX-2) and inducible nitric oxide synthase (iNOS), may be useful for treating inflammatory disorders [8]. Many compounds isolated from natural products reportedly show anti-inflammatory activity [9].

**Fig. 1:**
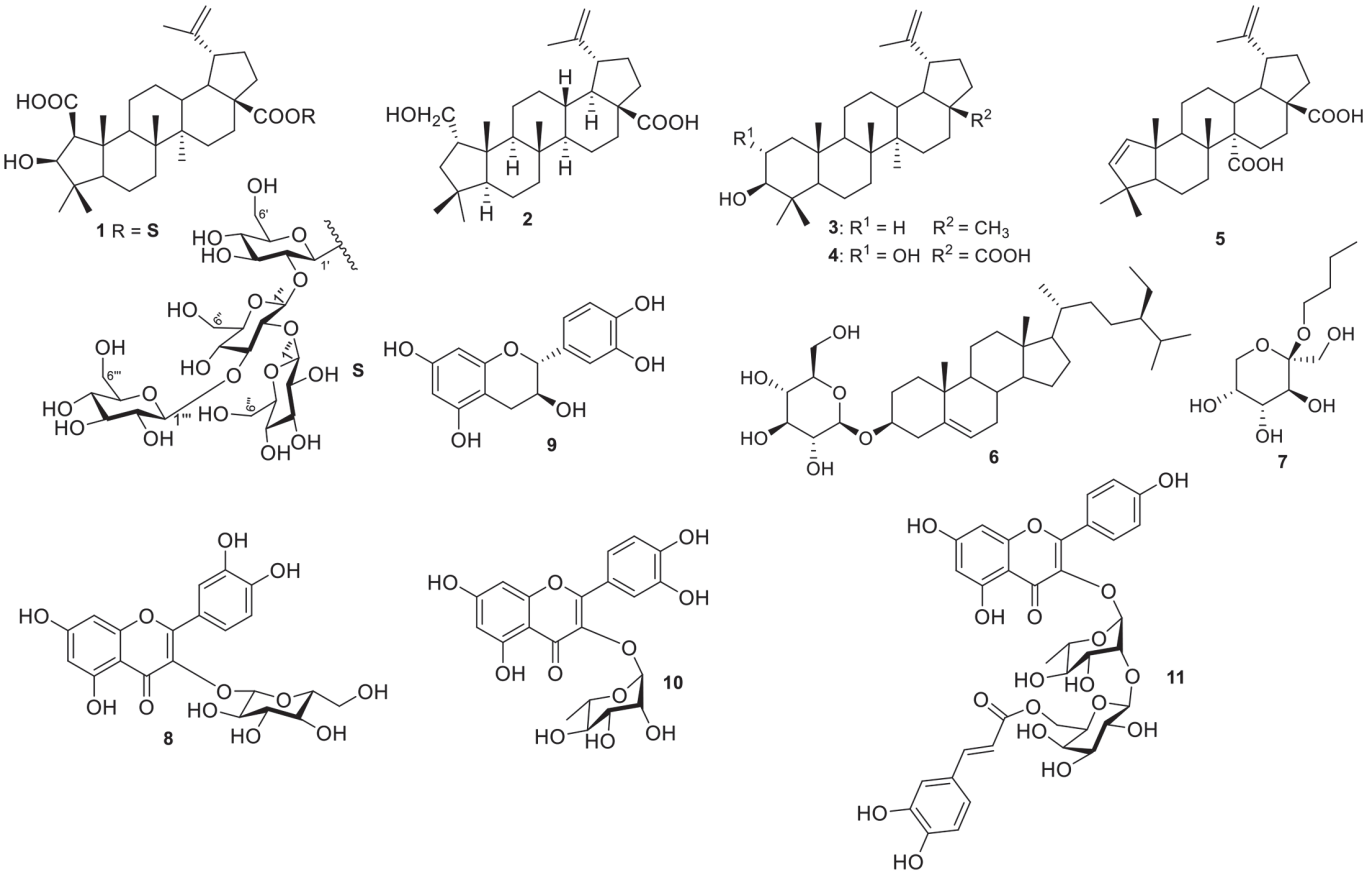
Structures of the isolated compound (1-11) purified from *G. leptostachya* aerial parts.

*Gouania leptostachya* DC. var. *tonkinensis* Pit., belonging to the family Rhamnaceae, is a traditional medicine used in Vietnam for the management of diverse ailments, such as inflammation, pain, and swelling [10]. The methanol extract of *G. leptostachya* shows various pharmacological effects, containing anti-inflammatory [10], antibacterial [11], and antioxidant [12] properties. Phytochemical investigation has revealed that phenolics, saponins, steroids, and benzopyran derivatives are the main components of *G. leptostachya* [13]. Previously, we reported four new dammarane triterpenoid saponins (gouaniasines VII–IX) from *G. leptostachya*. Moreover, their potential nitric oxide inhibitory activity was also evaluated [14]. However, the underlying mechanisms of inflammation have not yet been discovered. With the goal of identifying natural bioactive substances from *G. leptostachya* and elucidating their structures, in this study we describe the purification and structural identification of one unreported ceanothane-type triterpenoid glycoside (1), and 10 known metabolites (2-11) from *G. leptostachya* aerial parts (Fig. 1). The mechanisms underlying the anti-inflammatory properties of newly isolated compound **1** were also evaluated. Additionally, the potential anti-inflammatory properties of extract and fractions from *G. leptostachya* were the first time reported. Our findings enhance our comprehension of the secondary compounds synthesized by *G. leptostachya* and offer a logical basis for conducting additional research on the anti-inflammatory properties of this valuable medicinal herb.

## Material and Methods

### General Experimental Procedures

The Jasco P-1020 polarimeter was used to record optical rotation values, while the JASCO Report 100 infrared spectrophotometer was used to obtain FT-IR spectra. The Bruker Avance III 500 spectrometers were utilized to measure all NMR spectra, with TMS serving as the internal standard. The HR-ESI-MS were obtained from an Agilent 6530 Accurate-Mass Q-TOF LC/MS system. Silica gel (70-230, 230-400 mesh, Merck, USA) and YMC RP-18 resins (75 μm, Fuji Silysia Chemical Ltd., Japan) were used as adsorbents in the column chromatography. Merck KGaA (Germany) supplied the TLC plates (silica gel 60 F254 and RP-18 F254, 0.25 μm), which were detected under UV radiation (254 and 365 nm) and by spraying the plates with 10% H_2_SO_4_ followed by heating with a heat gun. All chemicals and reagents were procured from Sigma-Aldrich.

### Extraction and Isolation

The dried aerial parts of *G. leptostachya*, weighing 1.5 kg, were subjected to extraction with 96% EtOH, which was carried out thrice, using 10 L of solvent each time. The extraction was performed overnight at 50°C. The solvent was then evaporated under reduced pressure, which resulted in the formation of a tarry residue of EtOH weighing 148.0 g. This residue was then resuspended in H_2_O and partitioned successively with *n*-hexane, EtOAc, and *n*-BuOH, leading to the isolation of an *n*-hexane extract weighing 18.5 g, an EtOAc extract weighing 36.6 g, an *n*-BuOH extract weighing 21.0 g, and a water layer, which were obtained after removing the respective solvents. The obtained extracts can be further purified and characterized for the identification of the bioactive compounds present in the plant material. The detailed isolation process is described in the Supplementary data.

Physical and spectroscopic data of new compound: Gouanioside A (1). White shapeless material,[α]_D_^20^ 0.1, methanol). The major absorption band in the IR spectrum of 1: ν_max_ 3394, 2984, 1728, 1064, and 1049 cm^-1^. The detailed NMR data were described at Table 1; HR-ESI-MS *m/z* 1133.5385 [M-H]^-^ (calcd for C_54_H_85_O_25_^-^, 1133.5393), and *m/z* 1152.5779 [M+NH_4_]^+^ (calcd for C_54_H_90_NO_25_^+^, 1152.5796).

### Plant Material

In November 2015, the aerial portions of *G. leptostachya* were gathered from Thai Nguyen province, Vietnam and underwent taxonomic identification by Professor Pham Thanh Huyen. The corresponding voucher specimen (TB 10663C) has been stored at the Herbarium of NIMM (Vietnam).

### Cell Culture, NO and MTT Assay

The method for inhibiting the production of NO in murine macrophage RAW264.7 cells activated with LPS by the compound was conducted according to the literature [26, 28]. In summary, RAW264.7 cells, procured from American Type Culture Collection (ATCC, TIB-71, USA), were cultivated in DMEM enriched with 10% fetal bovine serum (FBS, 16000-044, Gibco, USA) and 100 U/ml penicillin-streptomycin (Gibco) at 37°C in a CO2 incubator. The detailed method is described in Supplementary information.

### Measure the Levels of PGE_2_ and TNF-α, an ELISA Assay

In order to determine the levels of PGE_2_ and TNF-α produced by RAW264.7 cells in response to LPS stimulation, an enzyme-linked immunosorbent assay (ELISA) was employed using a kit from R&D Systems (USA). RAW264.7 cells were initially cultured in a 6-well plate at a density of 2 × 10^6^ cells/well for 24 h and were subsequently stimulated with LPS for 12 h after pre-treatment with compound **1** for 1 h. The ELISA assay was conducted according to the manufacturer's instructions.

### Western Blot Analysis

The mechanism inflammatory of **1** was conducted to examine the capacity for influencing the expression of COX-2 mRNA using the RT-PCR technique, and assessing COX-2 gene activity through luciferase gene assay, as well as evaluating COX-2 protein expression through Western Blot analysis employing a method described previously [8, 28, 29]. RAW264.7 cells were seeded in 6-well culture plates at a density of 2 × 10^6^ cells/well and treated with lipopolysaccharide (LPS) at a concentration of 1 ng/ml for 16 h, following a pre-incubation with substance **1** for 1 h. The cells' lysate was obtained using a Cell Lysis Buffer from Cell Signaling Technology, following the manufacturer's instructions.

### Statistical Analysis

The statistical analysis was performed using the GraphPad Prism 8 software (GraphPad Software, USA). The differences between the group treated with the compound and the LPS-only group were analyzed using one-way analysis of variance (ANOVA) followed by Dunnett’s test. The level of significance was set at *p* < 0.01, ***p < 0.001.

### Molecular Docking Studies

Molecular docking investigations were conducted by the software AutoDock Vina 1.1.2 following the previously reported publication [39]. In brief, the crystal structure of COX-2 (PDB code, 1PXX) was retrieved from the RCSB Protein Data Bank. The ligands' three-dimensional (3D) structures were prepared utilizing Chem3D Pro and saved as mol files, and the most stable conformer was chosen for the docking study. The protein was prepared by removing water, initial ligands, repairing missing residues, and adding polar hydrogen atoms. The detailed procedure is shown in the Supplementary information.

## Results and Discussion

Dry samples of *G. leptostachya* aerial parts (1.5 kg) were extracted with pure ethanol (10 L × three times). A rotary evaporator was used to remove the solvent, yielding an EtOH residue (148.0 g) that was divided into exyl hydride, ethyl acetate, and butyl alcohol to yield an exyl hydride (18.5 g), ethyl acetate (36.6 g), butyl alcohol (21.0 g), and water extracts, respectively. Bioassay-guided fractionation of the EtOH extract of *G. leptostachya*, employing various chromatography separation techniques (silica gel; YMC RP-18, and Sephadex LH-20) resulted in the purification of a new saponin (**1**) and 11 known compounds (2-11, Fig. 1), namely epigouanic acid A (2)[14], lupeol (3) [15], alphitolic acid (4) [16], ceanothenic acid (5) [17], daucosterol (6) [18], and *n*-butyl-*β*-D-fructopyranoside (7) [19], quercitrin (8) [20], catechin (9) [21], isoquercitrin (10) [22], and kaempferol-3-*O*-(6-*O*-*E*-caffeoyl)-*β*-D-galactopyranosyl-(1→2)-*α*-L-rhamnopyranosid (11) [23]. The identified compounds were validated by matching their nuclear magnetic resonance (NMR) data with the relevant literature.

**Fig. 2:**
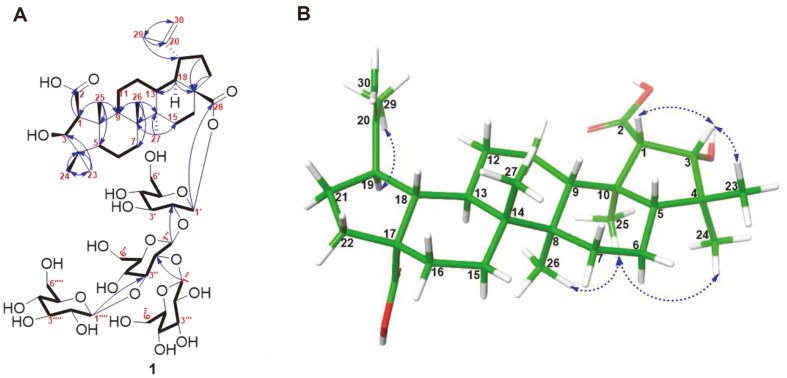
(**A**) Connectivity deduced by the COSY (bold), DEPT, and HSQC spectra, and key HMBC correlations (blue arrows) of **1**. (**B**) Significant NOESY (→) correlations of aglycon of **1**. After energy minimization, the threedimensional conformation of substance **1** was generated using the Macromodel program (version 12.5, Schrodinger LLC).

Substance 1 was a white shapeless material, with α_D_^20^ = −8.8 (c 0.1, MeOH) and a molecular formula of C_54_H_86_O_25_, as verified by both ionization modes of HR-ESI-MS, with a deprotonated molecular ion at *m/z* 1133.5385 [M-H]^-^ (calcd for C_54_H_85_O_25_^-^, 1133.5393) and quasi-molecular ion at *m/z* 1152.5779 [M+NH_4_]^+^ (calcd for C_54_H_90_NO_25_^+^, 1152.5796). The infrared (IR) spectra displayed strong signals at 3,394 and 1,728 cm^−1^, supporting the presence and functionalities of ester and hydroxyl groups. The ^1^H-NMR data of 1 indicated six methyl resonances at δ_H_: 0.92 (3H, s, H-24), 0.99 (3H, s, H-26), 1.02 (3H, s, H-27), 1.04 (3H, s, H-23), 1.19 (3H, s, H-25), and 1.71 (3H, s, H-29); The signals detected in the heteronuclear single quantum coherence (HSQC) spectrum exhibited associations with the carbon signals that corresponded to them, δ_C_ of 19.7, 17.6, 15.2, 32.2, 14.5, and 19.5, respectively. Additionally, four sugar proton signals at the anomeric position were detected at δ_H_ 4.59 (d, *J* = 7.5 Hz, H-1''''), 4.74 (d, *J* = 8.0 Hz, H-1'''), 5.05 (d, *J* = 7.5 Hz, H-1''), and 5.62 (d, *J* = 8.0 Hz, H-1')(Table 1). By utilizing carbon-13 nuclear magnetic resonance (^13^C-NMR) and heteronuclear single quantum coherence (HSQC) measurements together, a total of 30 signals for the aglycone and 24 signals for the sugar moiety were identified in the resulting spectra. The aglycon of 1 was identified as a ceanothane-type triterpenoid [24], using a comprehensive examination of the ^1^H- and ^13^C-NMR data (Table 1). The ^1^H-^1^H correlated spectroscopy (COSY) data indicated correlations among H-1/H3, H5/H6/H7, H9/H11/H12/H13/H18/H19/H21/H22, and H15/H16 (Fig. S3 and S19). The HMBC cross-peak from H-1 (2.37) to C-2 (177.0), H-23 (1.04) to C-3 (83.6), and H-29 (1.71), as well as H-30 (4.62, and 4.74) to C-19 (48.4), led to the identification of aglycon of 1 as epiceanothic acid. Furthermore, the acid hydrolysis of 1 was used to establish the definite configuration of the sugar moieties. The stereochemistry of the sugar component of 1 was characterized as *β*-D-glucose (Supplementary Data) [25, 26]; additionally, observation of cross-peaks between δ_H_ 5.62 (H-1', Glc-1) and δ_C_ 176.1 (C-28), δ_H_ 5.05 (H-1'', Glc-2) and δ_C_ 77.8 (Glc-1, C-2'), δ_H_ 4.74 (H-1''', Glc-3) and δ_C_ 82.6 (Glc-2, C-2''), and δ_H_ 4.59 (H-1'''', Glc-4) and δ_C_ 87.6 (Glc-2, C-3''') indicated that the *β*-D-glucopyranosyl-(1→3)-[*β*-D-glucopyranosyl -(1 → 2)]-*β*-D-glucopyranosyl -(1 → 2)-*β*-D-glucopyranosyl moiety was linked to the aglycon C-28 (Table 1). Further clarification of the proton signals corresponding to the sugar moiety of 1 was achieved based on ^1^H-^1^H COSY, TOCSY, and HMBC correlations (Supplementary Data). The NMR data of ^1^H and ^13^C indicated a similarity in the relative configuration of 1 with epiceanothic acid [24]. This was supported by the experimental results of nuclear Overhauser effect spectroscopy. Indeed, the correlations between H-1 (δ_H_ 2.37), H-3 (δ_H_ 4.10), and H-23 (δ_H_ 1.04) revealed that both H-1 and H-3 are *α*-oriented (Fig. 2B). Similarly, the connection among H-19 (δ_H_ 3.01), and H-29 (δ_H_ 1.71), and the absence of association between H-19 and H-18 suggested that H-19 is *β*-oriented (Fig. 2B). Consequently, the structure of new compound **1** was unambiguously identified as epiceanothic acid 28-*O*-*β*-D-glucopyranosyl-(1→3)-[*β*-D-glucopyranosyl-(1→2)]-*β*-D-glucopyranosyl-(1→2)-*β*-D-glucopyranoside, and named gouanioside A.

**Fig. 3:**
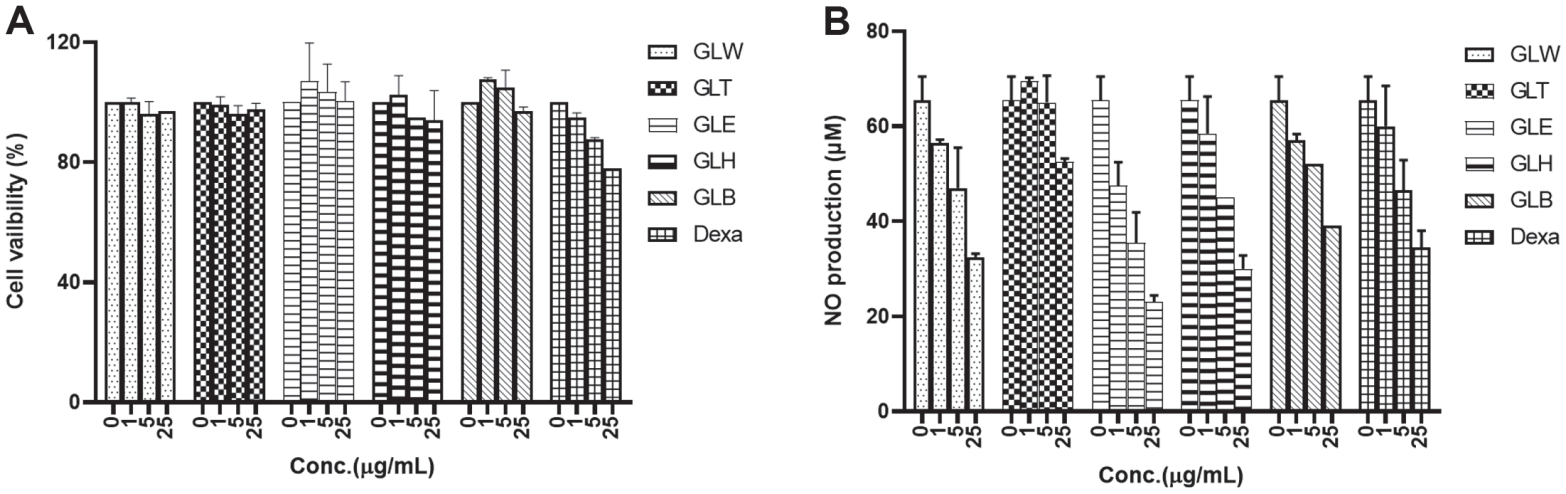
(**A**) Cytotoxic effects of ethanol (EtOH) extract (1, 5, and 25 μg) and fractions on RAW 264.7 cells over 3 days. GLT (EtOH extract), GLH (*n*-hexane fraction), GLB (BuOH fraction), GLE (EtOAc fraction), and GLW (water fraction), respectively. The positive control utilized in the experiment was Dexamethasone (Dexa). (**B**) Inhibition of the activation of RAW 264.7 cells by the EtOH extract and fractions in vitro. Different concentrations of the treatment were administered to the cells (1, 5, and 25 μg) of extract and fractions, or with Dexa as the positive control. The levels of nitric oxide (NO) in the culture supernatants were determined using the Griess method, **p < 0.01, ***p < 0.001. Dexamethasone was shown to have stable activity in the experiment.

For thousands of years, compounds isolated from natural products have served as powerful alternative treatments and pharmaceuticals [27]. Many inflammatory inhibitors are purified from both medicinal herbs and marine organisms. In particular, the medicinal plants *Ecklonia cava*, *Momordica charantia*, and *Gymnema sylvestre* have been widely used in the treatment of diabetes, showing efficacy, nontoxicity, and few to no side effects [28]. In our continuing search for new anti-inflammatory inhibitors from natural products, the anti-inflammatory properties of *G. leptostachya* crude EtOH extract, *n*-hexane fraction, ethyl acetate (EtOAc) fraction, *n*-BuOH fraction, and aqueous fractions were evaluated in terms of their inhibitory effects on the lipopolysaccharide (LPS)-induced expression of NO production. To avoid cytotoxicity, the effects of the crude extract (EtOH) and fractions on RAW 264.7 cells were examined at concentrations of 1, 5, and 25 μM over 3 days using the MTT method [8]. None of the tested crude extract concentrations or fractions showed significant cytotoxicity, *i.e.*, not even at 25 μM (Fig. 3A). NO is regarded as a pivotal factor in the pathogenesis of inflammation. Thus, NO concentrations were evaluated using the Griess method in this study [25]. All extracts and fractions of *G. leptostachya* exhibited anti-inflammatory effects, in a concentration-dependent manner (Fig. 3B).

To avoid cytotoxic effects of the new compound 1, the MTT method was also applied. The results showed no cytotoxicity of new compound 1, even at 25 μM (Fig. S10, Supporting Data). To evaluate the inhibitory properties of new compound 1 on the LPS-induced production of NO in RAW264.7 cells, NO was measured using the Griess reaction.

**Fig. 4:**
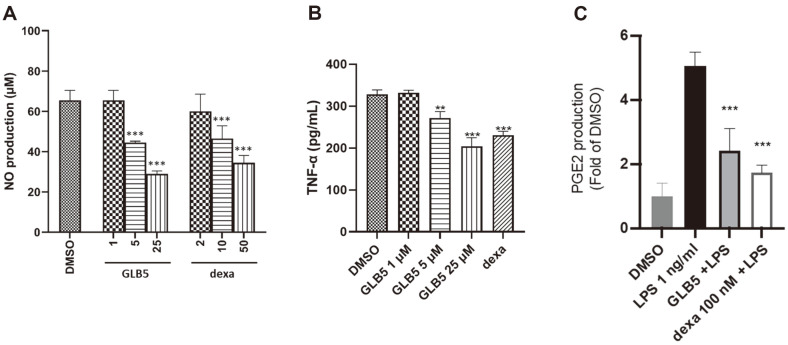
Inhibitory effects of new compound **1** (GLB5) on lipopolysaccharide (LPS)-stimulated (**A**) NO, (**B**) PGE_2_, and (**C**) TNF-α production in RAW264.7 macrophages, respectively. RAW264.7 cells (2 × 10^6^ cell/well) were pretreated with compound **1** (GLB5) for 1 h before being stimulated for 12 h with LPS. The NO, PGE_2_, and TNF-α levels were determined by the Griess method and an enzyme linked immunosorbent assay kit. Data represent five independent experiments and are expressed as the mean ± the standard error of the mean (SEM). Dexamethasone (dexa) was used as the positive control. The experiments were repeated five times. (**p < 0.01, ***p < 0.001). Dexamethasone was shown to have stable activity in the experiment.

**Fig. 5:**
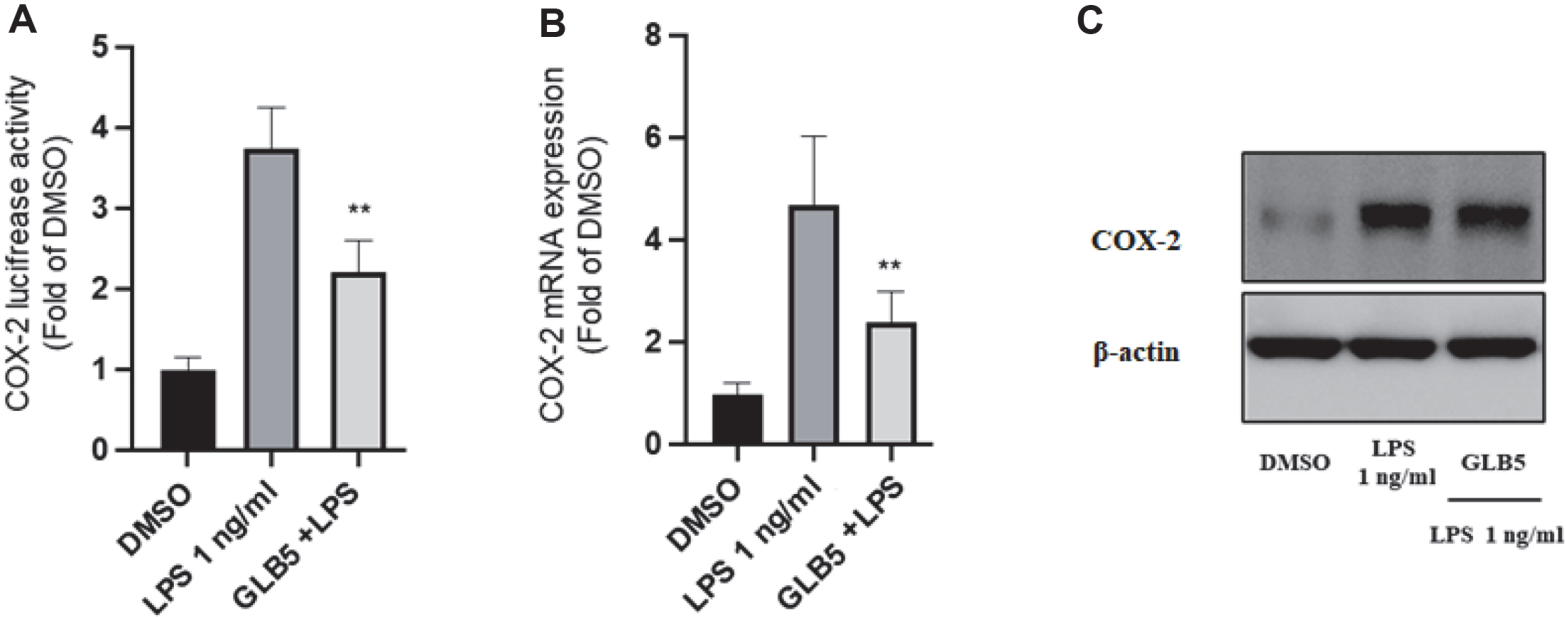
(**A**) Effects of compound **1** (GLB5, 10 μM) with the capacity for influencing the expression of COX-2 mRNA using the RT-PCR technique, (**B**) COX-2 gene activity through luciferase gene assay, and (**C**) evaluating COX-2 protein expression through Western blot analysis, respectively. β-actin as a loading control. The intensity of the bands was measured using ImageJ software. **p < 0.01 versus the LPS alone group.

In order to evaluate its effect on NO production, further tests on the anti-inflammatory activities of 1 were carried out by ELISA method. The effects of 1 on mRNA expression levels of pro-inflammatory cytokines (PGE_2_ and TNF-α) and COX-2 protein were determined in LPS-induced RAW264.7 cell lines using ELISA and Western blot methods. To determine the inhibitory effects of pro-inflammatory cytokines on the expression of PGE_2_ and TNF-α, LPS-stimulated RAW264.7 cells were treated with compound 1. The experiments were carried out using enzyme-linked immunosorbent assay kits. As shown in Figs. 4A and 4B, 1, 1 showed potent inhibitory effects on both NO and TNF-α, in a concentration-dependent manner. Additionally, 1 reduced TNF-α cell expression at a concentration of 10 μM. The results suggest that 1 significantly inhibits pro-inflammatory cytokines. NO-induced COX-2 expression is mediated partially by the cyclic guanosine monophosphate (cGMP)-dependent pathway. Saponins showed anti-inflammatory effects via regulation of iNOS, COX-2, and other inflammatory factors [28]. The mechanism inflammatory of 1 was conducted to examine the capacity for influencing the expression of COX-2 mRNA using the RT-PCR technique, and assessing COX-2 gene activity through luciferase gene assay, as well as evaluating COX-2 protein expression through Western Blot analysis employing a method described previously [8, 29]. Compound 1 exhibits inhibitory activity against COX-2 luciferase activity (Fig. 5A). Compound 1 demonstrates a significant pharmacological effect in modulating the expression of COX-2 mRNA, resulting in a notable reduction of its cellular abundance (Fig. 5B). Additionally, Compound 1 (10 μM) demonstrated a significant ability to suppress the expression of COX-2 in LPS-activated cells. Therefore, the results suggest that compound **1** exerts an inhibitory effect on the biosynthesis of pro-inflammatory cytokines in macrophages that have been stimulated to an activated state.

**Fig. 6:**
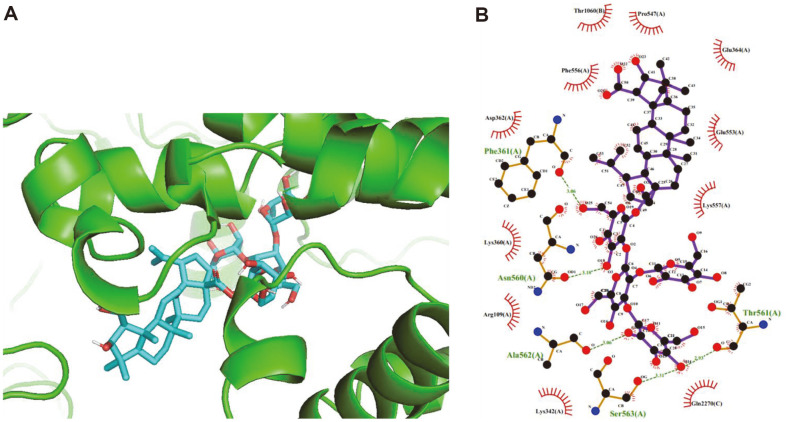
Molecular docking results of compound **1** with the COX-2 protein (PDB: 1PXX). (**A**) three-dimensional (3D) docking image of compound **1** was created by Pymol 2.5. (**B**) two-dimensional (2D) docking image of **1** was created by LigPlot+ 2.2.

To further identify the anti-inflammatory properties of new compound **1** against COX-2 protein expression, molecular docking studies were carried out on 1 and COX-2 protein (PDB ID, 1PXX, Protein Data Bank). The procedure is shown in the Supporting material. The results showed that 1 could bind tightly to the catalytic amino acid residues to inhibit COX-2 protein expression. Compound 1 interacted with catalytic residues for binding, *i.e.*, Phe361(3.0 Å), Asn560(3.18 Å), Ala562(3.06 Å), Ser563(3.31 Å), and Thr561(2.93 Å) (Figs. 6A and 6B). Additionally, compound **1** binds to the active site of COX-2 with binding affinities of –6.3 kcal/mol. The results suggested that the sugar moiety in the chemical structure of saponin could be a crucial factor in inhibiting the COX-2 enzyme. Therefore, the molecular docking simulations displayed strong interactions with COX-2 protein. Taken together, the results imply that compound **1** isolated from *G. leptostachya* may be useful for the treatment of inflammatory diseases.

Natural bioactive products play an important role in controlling the inflammatory response and are thus important in terms of drug discovery. Saponins, a class of secondary metabolites, are widely distributed in various plant species and have been extensively studied for their potential medicinal plants [30] and marine organisms [31], demonstrate a diverse array of bioactive properties, including anticancer [32], anti-bacterial [33], antifungal [34], anti-inflammatory [32], and antioxidant [35] activities. Triterpene saponin is the main metabolite responsible for the pharmacological effects of *Gynostemma pentaphyllum* [36], *Astragalus membranaceus* [37], Korean red ginseng [29], and *Momordica charantia* [38] extracted from medicinal herbs. Our study showed that compound **1** strongly inhibits the production of NO in LPS-stimulated murine RAW254.7 macrophage cells. Moreover, exposure to 1 (10 μM) elicited a notable reduction in the secretion of PGE_2_ and TNF-α, along with a marked inhibition of COX-2 protein expression in RAW264.7 cells that were stimulated with LPS. The molecular docking simulation implied the possible mechanism of NO inhibition to be the interactions of a new ceanothane-type triterpenoid saponin with COX-2 protein. In conclusion, this study demonstrated that guanosine A (1), a new triterpene saponin found in the aerial parts of *G. leptostachya*, may be useful for facilitating the discovery of innovative anti-inflammatory therapeutics and treatments for inflammation-related diseases.

## Supplemental Materials

Supplementary data for this paper are available on-line only at http://jmb.or.kr.

## Figures and Tables

**Table 1:** ^1^H (500 MHz, CD_3_OD) and ^13^C-NMR (125 MHz, CD_3_OD) spectroscopic data, δ ppm, of compound **1**.

Position	1				
	δ_C_	δ_H_ (*J* in Hz)		δ_C_	δ_H_ (*J* in Hz)
Aglycon			Sugar		
1	62.6	2.37 d (7.5)	*Glc*		
2	177.0	-	1'	93.4	5.62 d (8.0)
3	83.6	4.10 d (7.0)	2'	77.8	3.87 m
4	43.7	-	3'	78.8	3.42 m
5	63.6	0.90 s	4'	70.8	3.45 m
6	19.0	1.37 m, 1.61 m	5'	78.3	3.80 m
7	35.7	1.35 m, 1.45 m	6'	62.3	3.72 m, 3.84 m
8	43.0	-	*Glc*		
9	51.9	1.52 m	1''	102.6	5.05 d (7.5)
10	49.5	-	2''	82.6	3.87 m
11	26.4	1.11 m, 1.68 m	3''	87.6	3.69 m
12	24.9	1.41 m, 1.52 *	4''	71.1	3.29 m
13	39.1	2.28 m	5''	78.2	3.40 m
14	43.8	-	6''	63.5	3.60 m, 3.93 m
15	32.1	1.10 m, 1.60 m	*Glc*		
16	32.5	1.40 m, 2.58 m	1'''	104.8	4.74 (d, 8.0)
17	57.9	-	2'''	75.9	3.24 m
18	50.7	1.62 t (11.0)	3'''	78.4	3.39 m *
19	48.4	3.01 m	4'''	71.4	3.34 m
20	151.8	-	5'''	77.5	3.40 m
21	31.4	1.39 m, 1.90 m	6'''	62.6	3.65 m, 3.90 m
22	37.5	1.50 m, 2.01 m	*Glc*		
23	32.2	1.04 s	1''''	104.8	4.59 d (7.5)
24	19.7	0.92 s	2''''	75.4	3.30 m
25	14.5	1.19 s	3''''	78.4	3.39 *
26	17.6	0.99 s	4''''	71.6	3.35 m
27	15.2	1.02 s	5''''	77.5	3.30 *
28	176.1	-	6''''	62.6	3.76 m, 3.99 m
29	19.5	1.71 s			
30	110.3	4.62 d (1.5), 4.74 s			

*Overlapped signals and chemical shifts may be interchanged

Assignments were determined by DEPT, COSY, HSQC, HMBC, TOCSY, and NOESY experiments.
