# Supplementary material for: A New Ceanothane-Type Triterpenoid Saponin Isolated from Gouania leptostachya DC. var. tonkinensis Pit. and Its Underlying Anti-Inflammatory Effects
Source: J Microbiol Biotechnol. 2023 Apr 24;33(7):941–8. doi: 10.4014/jmb.2301.01040 (PMC10394341; doi:10.4014/jmb.2301.01040)
Supplement: Supplementary file 1 [file jmb-33-7-941-supple.pdf]

SUPPLEMENTARY MATERIAL

A New Ceanothane-Type Triterpenoid Saponin Isolated from *Gouania leptostachya* DC. var. *tonkinensis* Pit. and Its Underlying Anti-Inflammatory Effects

Abstract

Metabolites from medicinal plants continue to hold significant value in the exploration and advancement of novel pharmaceuticals. In the search for plants containing compounds with anti-inflammatory effects, we observed that the ethanol (EtOH) extract obtained from the aerial components of *Gouania leptostachya* DC. var. *tonkinensis* Pit. exhibited substantial suppression of nitric oxide (NO) in vitro. In a phytochemical study on an EtOH extract of *G. leptostachya*, 11 compounds were purified, including one unreported compound namely gouanioside A (**1**). Their chemical structures were unambiguously determined through the use of various spectroscopic techniques, such as 1 and 2D NMR, IR, and HR-ESI-MS, and by producing derivatives via chemical reactions. The EtOH extract, fractions, and a new compound exerted inflammatory effects by altering NO synthesis in murine RAW264.7 macrophage cells stimulated with lipopolysaccharide. The underlying inflammatory mechanism of the new compound **1** was also explored through various in vitro experiments. The results of this study indicate the potential usefulness of new compound **1** from *G. leptostachya* as a treatment for inflammatory diseases.

Keywords: *Gouania leptostachya* · saponin· anti-inflammatory effect · gouanioside A

Acid hydrolysis and sugar identification

Compound **1** (3.0 mg) was dissolved in 1 mL of 1.0 N HCl (H_2O/dioxane, 1:1, v/v), and each solution was heated at 80°C for 8 h. After cooling, the solvent was evaporated under N_2, and the residue was separated by solvent-solvent partition using CH_2Cl_2 and water. The monosaccharide in hydrolysis products was purified by preparative TLC (CH_2Cl_2/MeOH/H_2O, 2:1:0.2, v/v/v) and then immediately measured optical rotation. The specific rotation ($[\alpha]_D^{20}$) of sugar was determined and compared to the literature. The presence of D-glucose in the acid hydrolysis products of compound **1** was confirmed by TLC analysis and comparison of their optical rotation to those of authentic D-glucose [R_f 0.30, $[\alpha]_D^{20}$ = +45.8 (*c* 0.1, H_2O)] as reported [1-3].

Extraction and isolation

The *n*-butanol fraction (20.0 g) was chromatographed on a silica gel column and eluted with a solvent system of ethyl acetate-methanol-water (6:1:0.2 → 2:1:0.2, v/v/v) to obtain five subfractions (B1–B5). Subfraction B3 (5.1 g) was separated via Diaion HP-20 column chromatography (CC) and successively eluted with water, EtOH (96%), and acetone to obtain two subfractions (B3.1 and B3.2). Subfraction B3.1 (3.5 g) was separated via reverse-phase CC with a C_{18} column with methanol-water (1:2 → 1:1, v/v) to obtain compounds **1** (7.0 mg), **2** (100 mg), **11** (12 mg), and **6** (12 mg) and eight subfractions (B3.1.1–B3.1.8). Subfraction B3.1.8 (0.5 g) was purified via silica gel CC with chloroform-methanol-water (3.8:1:0.1, v/v/v) as the mobile phase to obtain compounds **3** (36 mg) and **4** (21 mg).

The ethyl acetate fraction (35.0 g) was separated via silica gel CC with dichloromethane-methanol (30:1 → 5:1, v/v) as the gradient-elution solvent to obtain eight subfractions (E1–E8). E1 (4.7 g) was separated by silica gel CC using *n*-hexane-acetone (20:1, v/v) as the mobile phase to obtain compound **7** (1.0 g). Subfraction E2 (7.2 g) was separated into six subfractions (E2.1–

E2.6) via silica gel CC and elution with dichloromethane-methanol (40:1, v/v). Subfraction E2.3 (0.4 g) was separated via silica gel CC with *n*-hexane-ethyl acetate-methanol (6:1:0.4, v/v/v) as the mobile phase to obtain compounds **5** (10 mg), **9** (100 mg), and **10** (86 mg). Compound **8** (28 mg) was isolated from sub-fraction E2.6 (0.2 g) by silica gel CC with chloroform-methanol (25:1, v/v) as the mobile phase.

Cell culture, NO and MTT assay

RAW264.7 cells were obtained from American Type Culture Collection (ATCC, TIB-71, Rockville, MD, USA) and cultured in DMEM supplemented with 10% fetal bovine serum (FBS, 16000-044, Gibco, Grand Island, NY, USA) and 100 U/mL penicillin-streptomycin (Gibco) at 37 °C in incubator containing 5% CO2. Cells were plated at 5 × 104 cells/well in 96-well culture plates and then were incubated with compounds for 1 h before LPS (0.1 ng/mL) stimulation for 18 h. Cell supernatant (100 µL) was mixed with an equal amount of Griess reagent consisting of 1% sulfanilamide (Sigma, St. Louis, MO, USA) and 0.1% N-(1-naphthyl)ethylenediamine (Sigma) in 5% phosphoric acid. Its absorbance was measured at 550 nm using a microplate reader (Varioskan LUX, Thermo Fisher Scientific Inc., Waltham, MA, USA). Cell viability was evaluated by the MTT assay. Briefly, 3-(4,5-dimethylthiazol-2-yl)-2,5 diphenyl tetrazolium bromide (MTT, Sigma) was added to the cells for 2 h. The supernatant was removed and then the MTT formazan crystals were dissolved in dimethyl sulfoxide (DMSO, Sigma).

Molecular Docking Studies

Molecular docking studies were conducted using AutoDock Vina 1.1.2 [4]. The structure of COX-2 (PDB code, 1PXX) was obtained from the RCSB Protein Data Bank. The three-dimensional (3D) structures of the ligands were prepared using Chem3D Pro and saved as mol files. The most stable conformer was selected as a ligand for the docking study. The protein was

prepared by deleting water, removing initial ligands, repairing the missing residues, and adding polar hydrogen atoms. The grid box dimensions were set at 20 × 20 × 20 Å with a grid spacing of 0.5 Å and box center coordinates (x: 40.95, y: 33.77, z: 29.10) to locate the active site of the enzyme. Docking calculations were run by the Lamarckian genetic algorithm (run times of 50, population size of 300, and default setting for other miscellaneous parameters). The docked complex with the lowest binding energy was chosen to represent the most favorable interaction between the ligand and protein. The results from AutoDock were re-rendered by Pymol 2.5 and LigPlus v2.2.

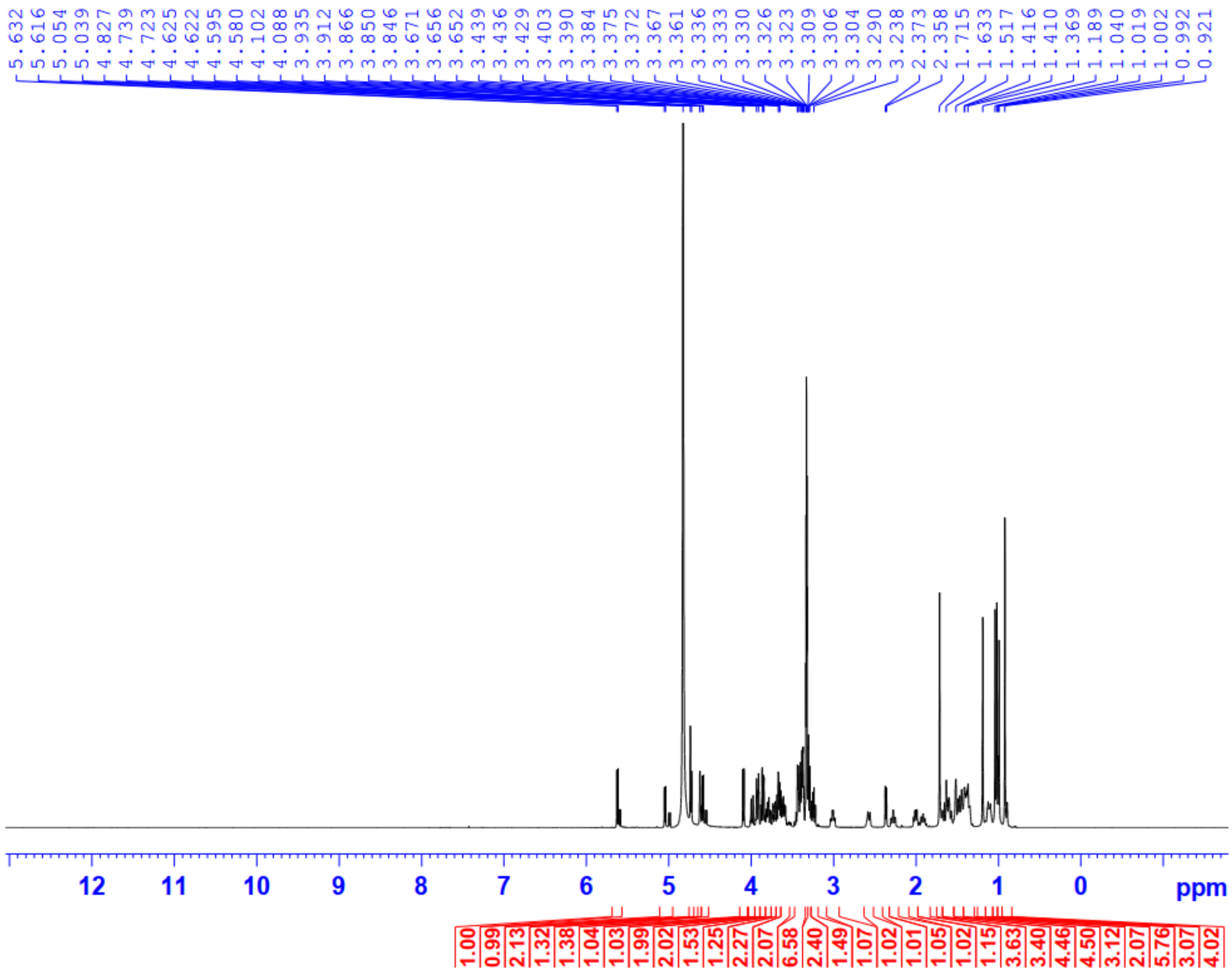

Figure S1. **^{1}H-NMR spectrum of 1 in CD_3OD (500 MHz)**

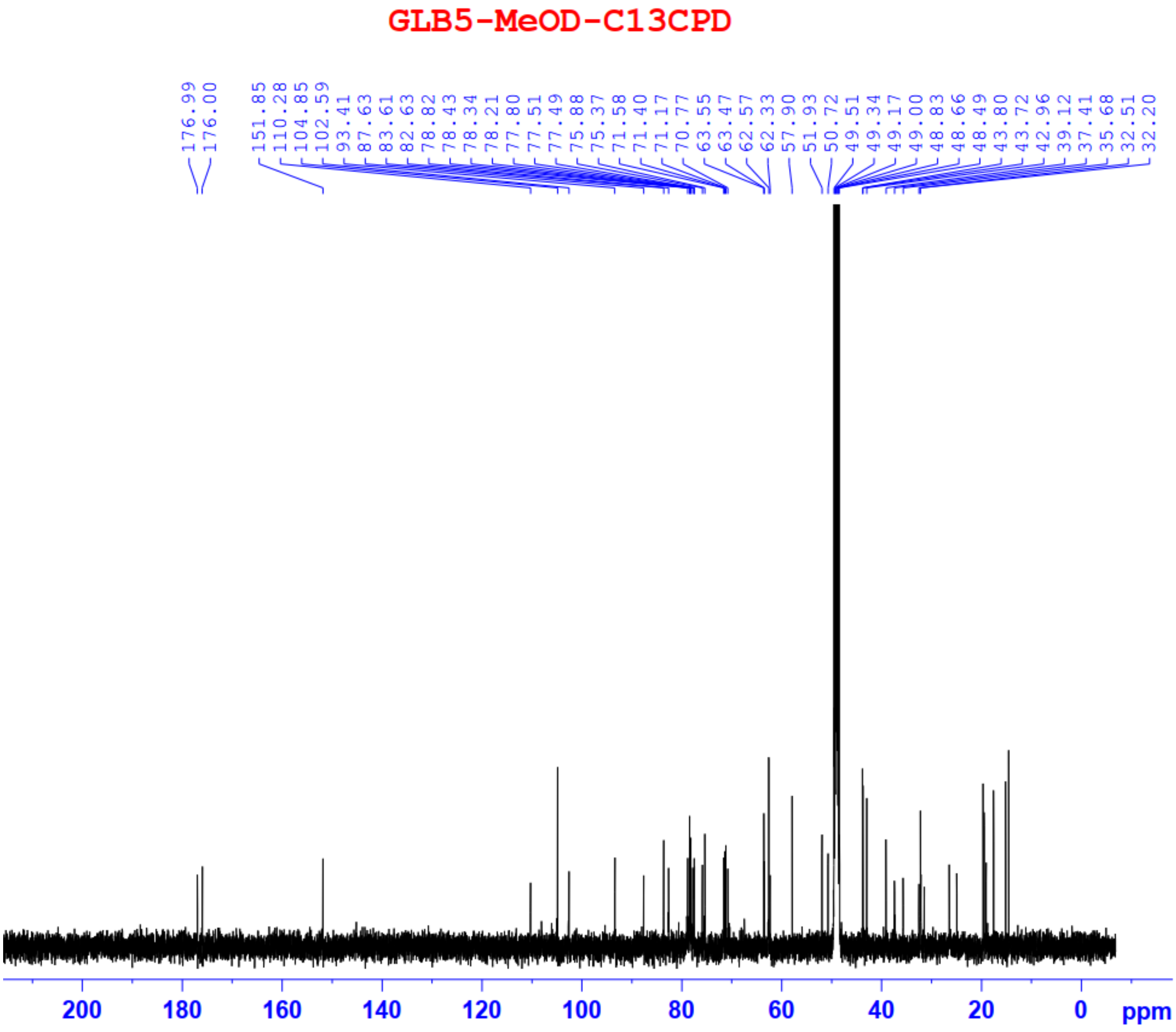

Figure S2. **^{13}C-NMR spectrum of 1 in CD_3OD (125 MHz)**

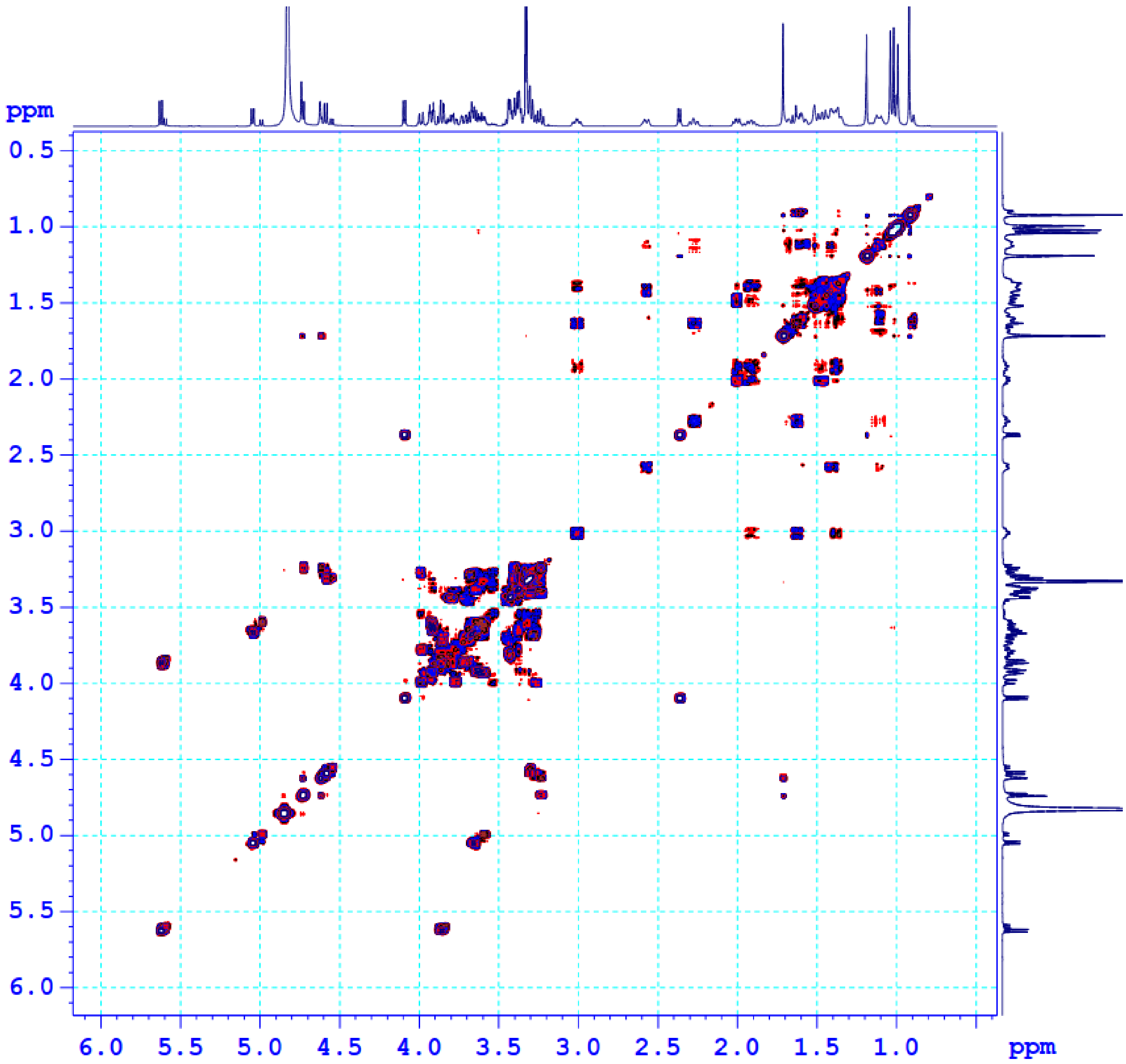

Figure S3. COSY spectrum of compound 1

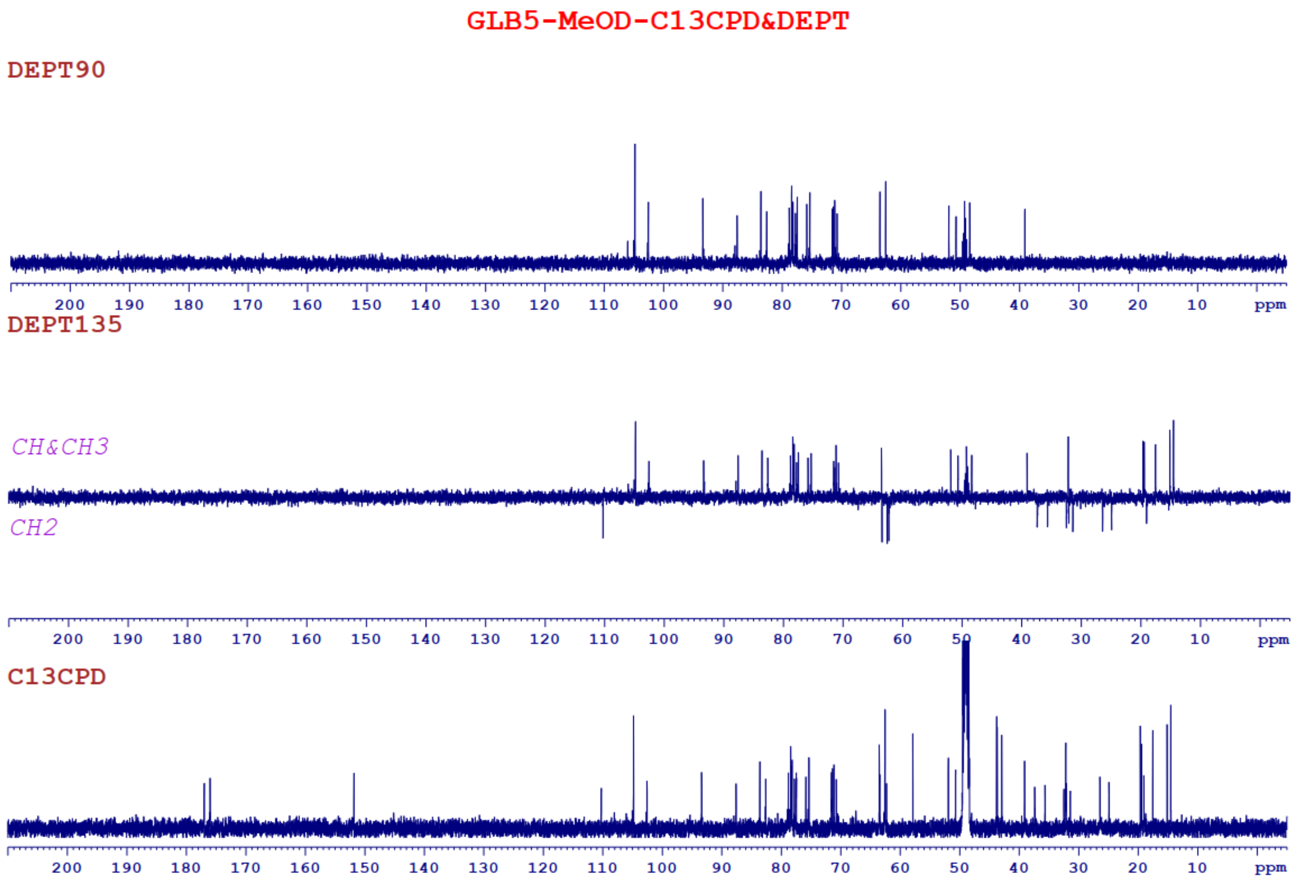

Figure S4. DEPT spectrum of compound 1

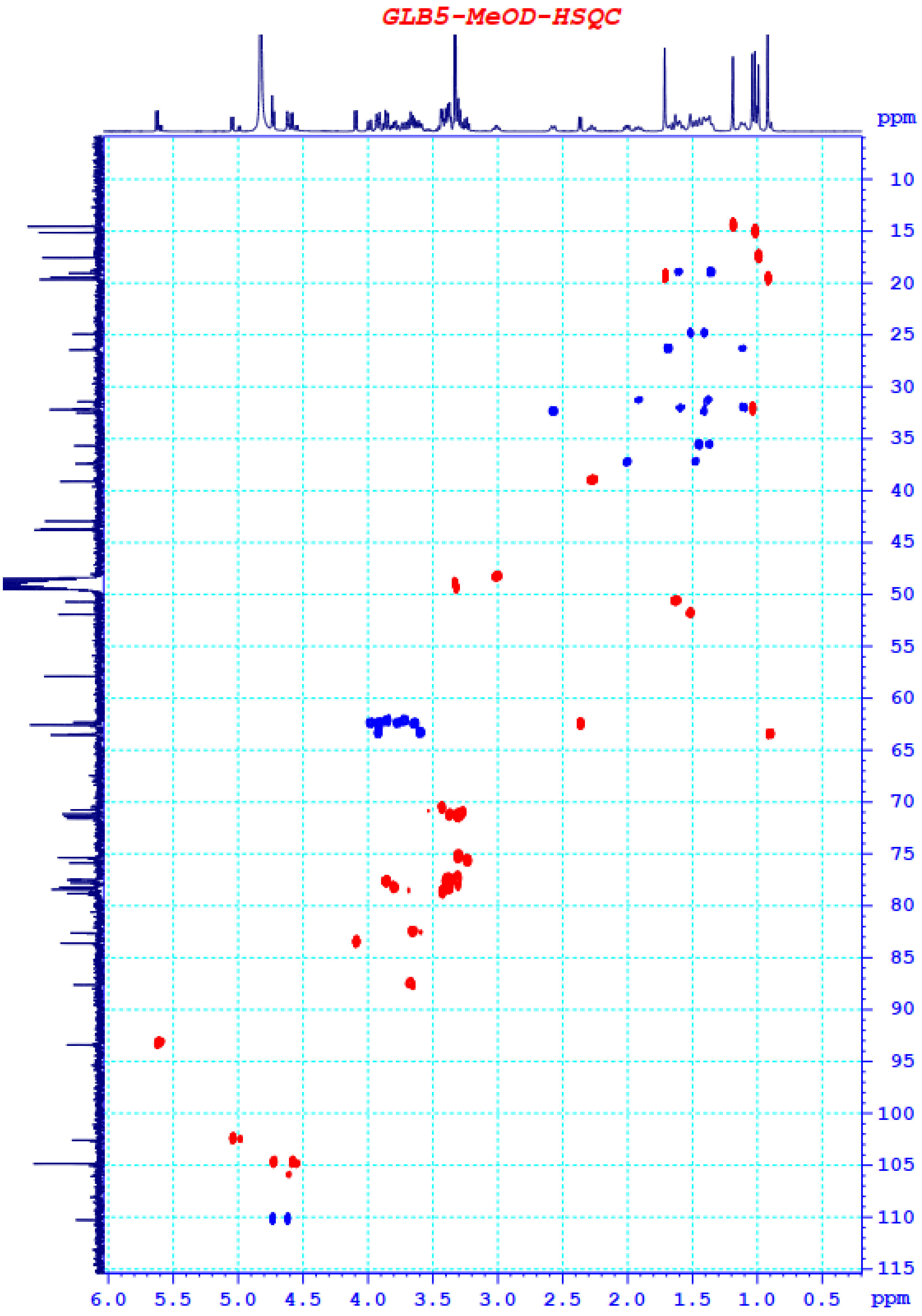

Figure S5. HSQC spectrum of compound 1

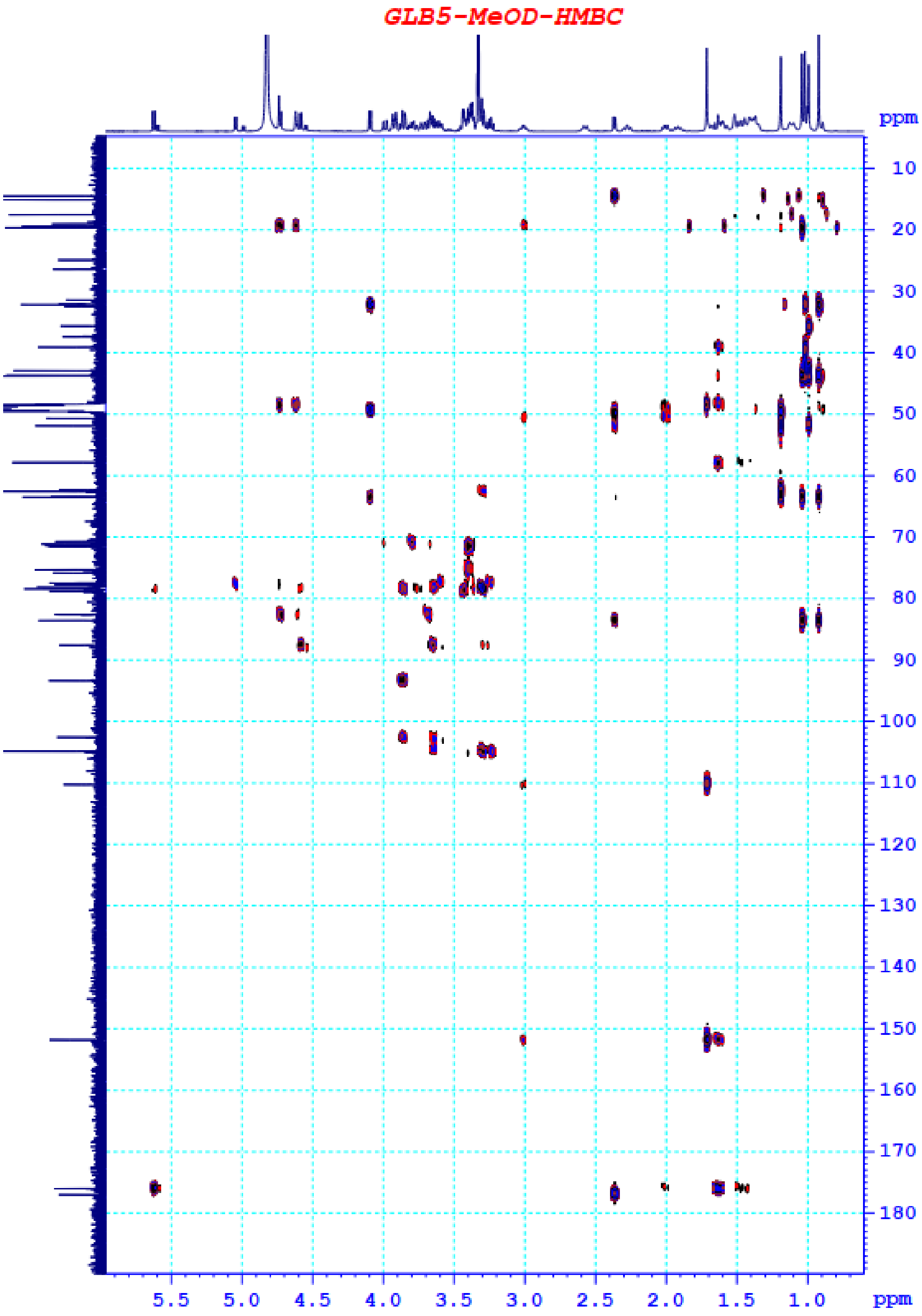

Figure S6. HMBC spectrum of compound 1

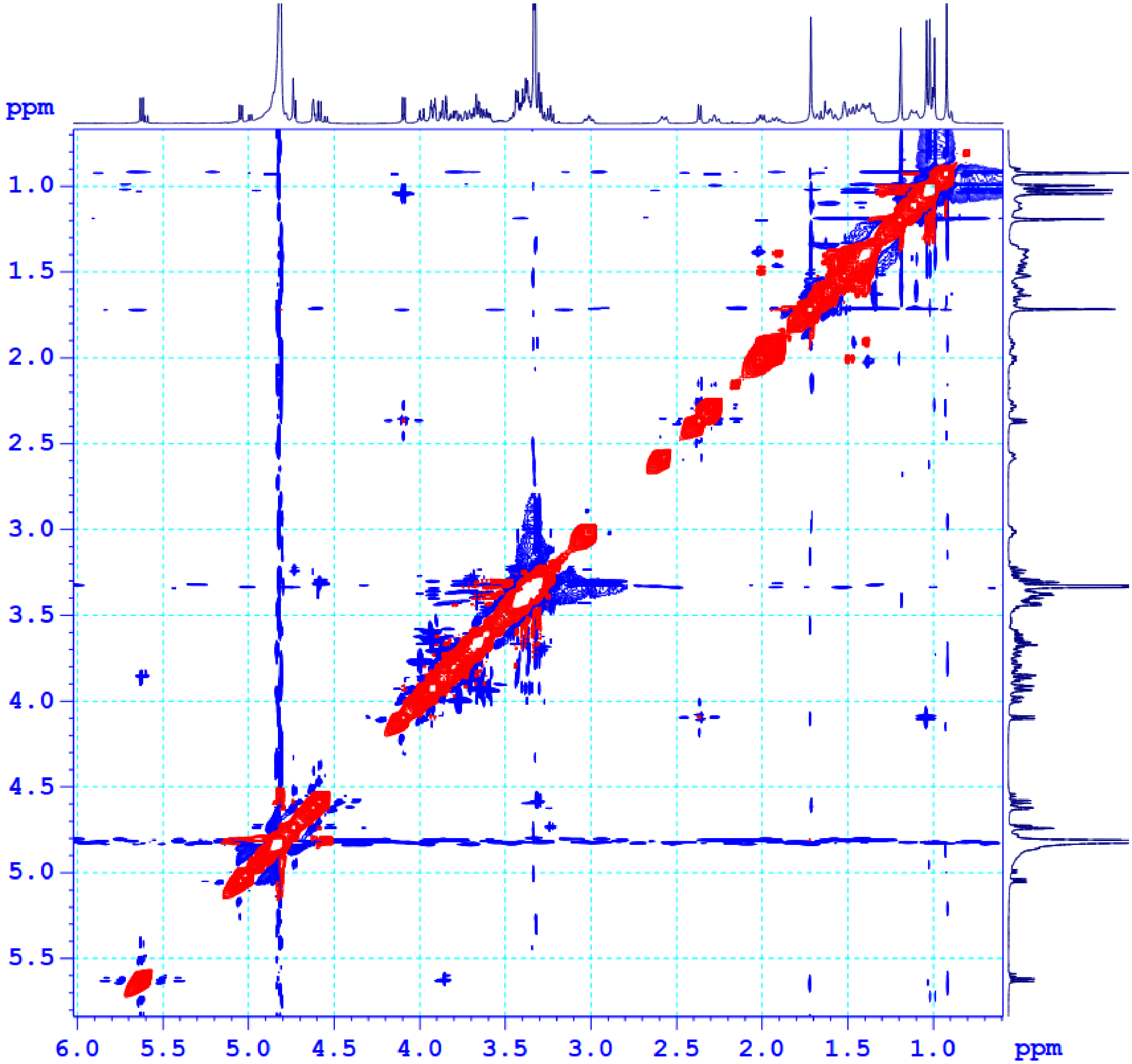

Figure S7. NOESY spectrum of compound 1

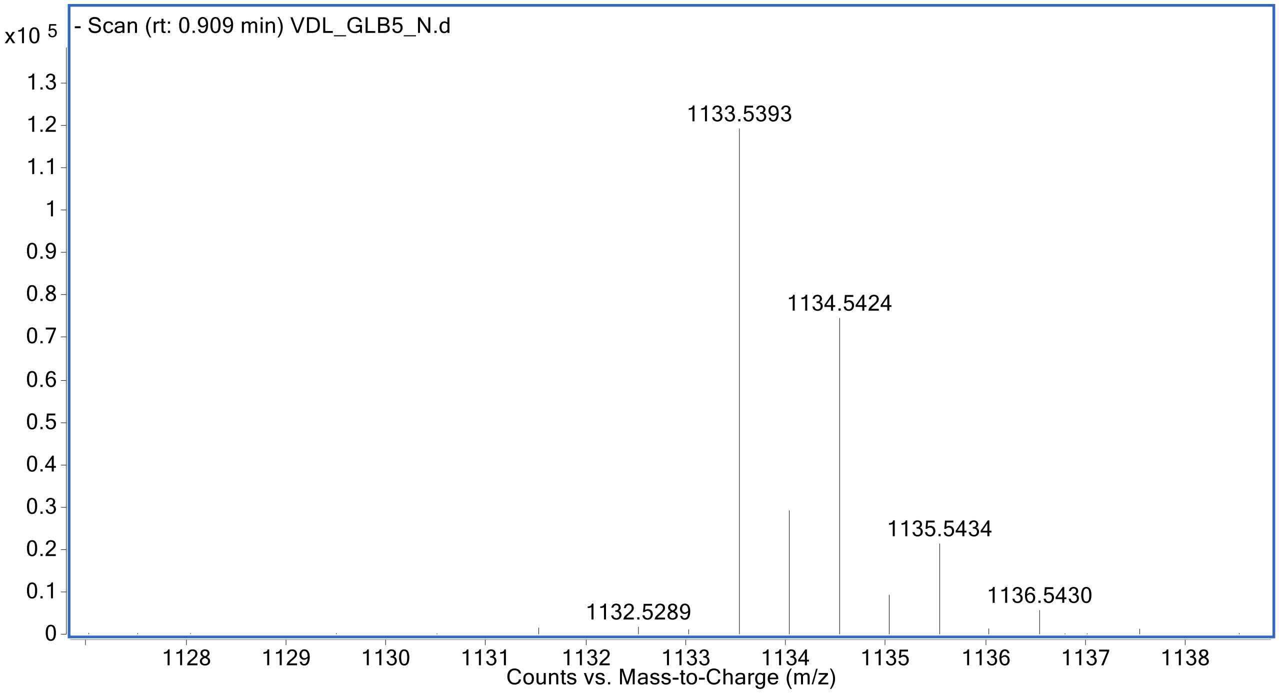

Figure S8. HR-ESI-MS spectrum of compound 1 (negative mode)

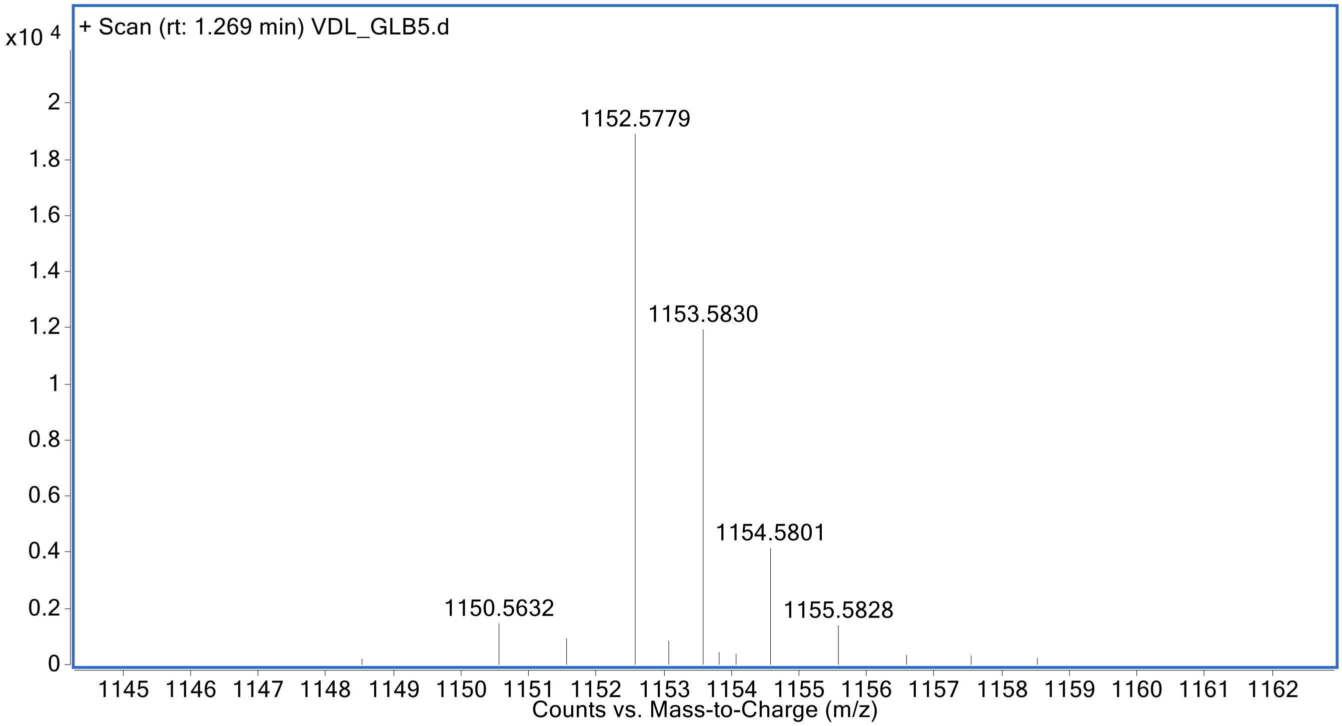

Figure S9. HR-ESI-MS spectrum of compound 1 (positive mode)

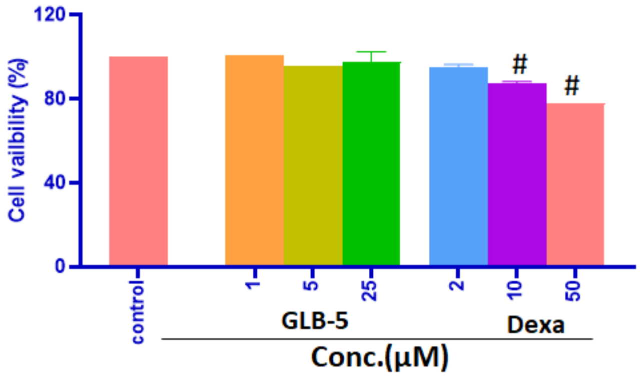

Figure S10. Cytotoxic properties of compound **1** (GLB5) RAW 264.7 cells after treatment with compound **1** (1, 5, and 25 μM, respectively) and dexamethasone (DEX) (2, 10, and 50 μM, respectively). Dexamethasone was used as the positive control.

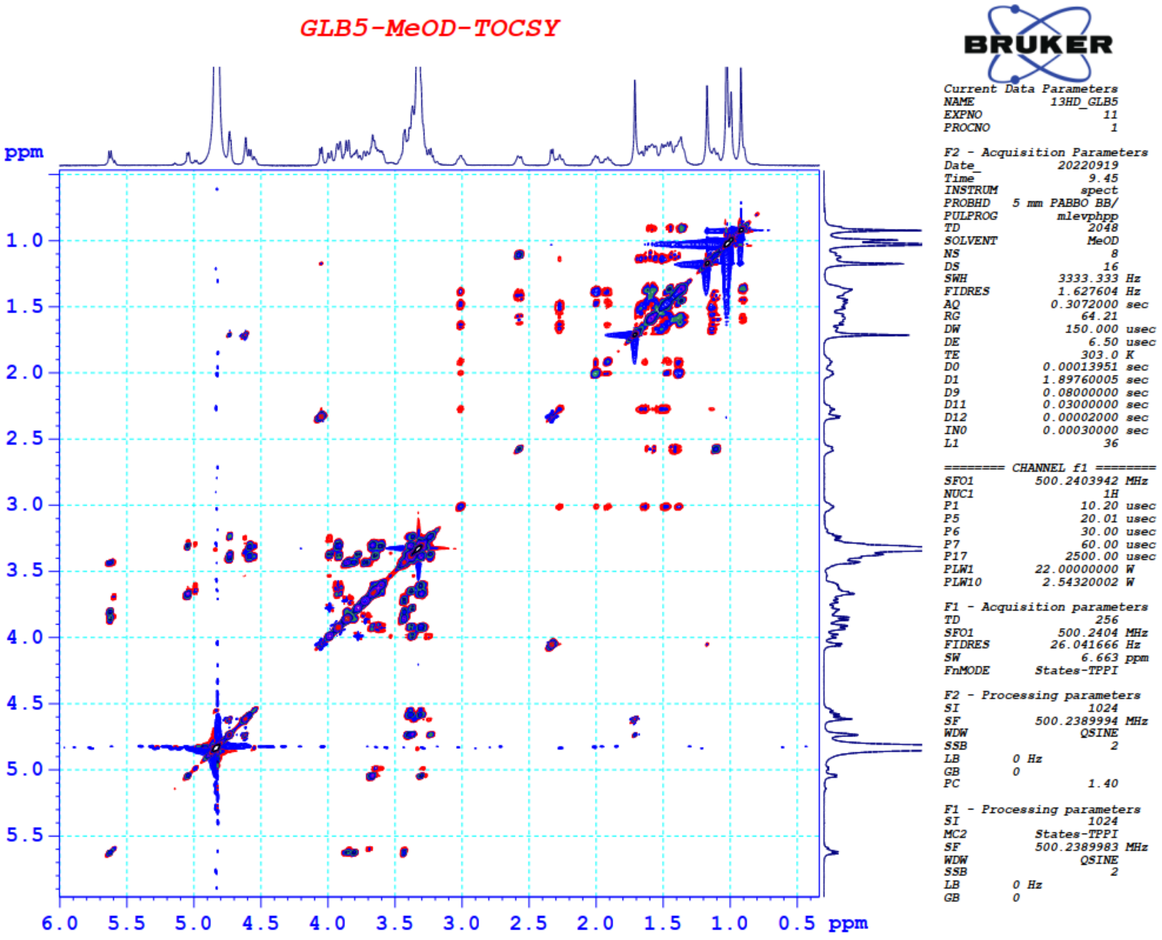

Figure S11. TOCSY spectrum of compound 1

Table S1. ^{1}H (500 MHz, CD_3OD) and ^{13}C NMR (125 MHz, CD_3OD) spectroscopic data of **1** and epiceanothic acid (Pyridine-d_5)

**C**	**Epiceanothic acid (2)** δ_C	**1**	
		δ_C	δ_H mult. (J = Hz)
Aglycon			
1	62.7	62.6	2.37 d (7.5)
2	178.8	177.0	-
3	83.1	83.6	4.10 d (7.0)
4	42.9	43.7	-
5	63.1	63.6	0.90 s
6	18.4	19.0	1.37 m, 1.61 m
7	34.8	35.7	1.35 m, 1.45 m
8	42.0	43.0	-
9	51.1	51.9	1.52 m
10	49.8	49.5	-
11	25.9	26.4	1.11 m, 1.68 m
12	24.6	24.9	1.41 m, 1.52 *
13	38.5	39.1	2.28 m
14	43.1	43.8	-
15	31.2	32.1	1.10 m, 1.60 m
16	33.0	32.5	1.40 m, 2.58 m
17	56.5	57.9	-
18	49.8	50.7	1.62 t (11.0)
19	48.1	48.4	3.01 m
20	151.1	151.8	-
21	30.5	31.4	1.39 m, 1.90 m
22	37.6	37.5	1.50 m, 2.01 m
23	32.1	32.2	1.04 s
24	19.9	19.7	0.92 s
25	14.6	14.5	1.19 s
26	16.9	17.6	0.99 s
27	15.0	15.2	1.02 s
28	175.1	176.1	-
29	110.0	19.5	1.71 s
30	19.5	110.3	4.62 d (1.5), 4.74 s

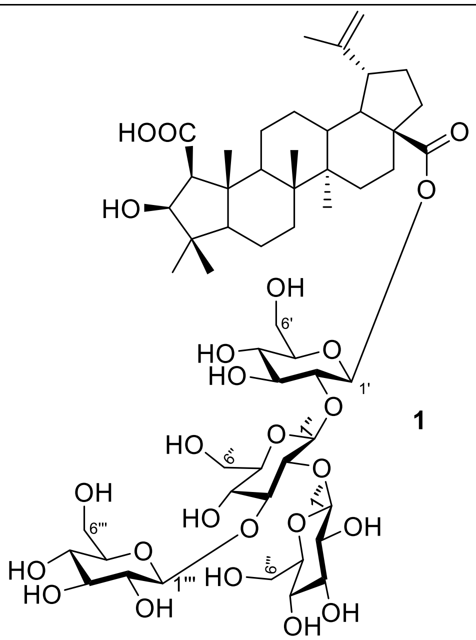

1

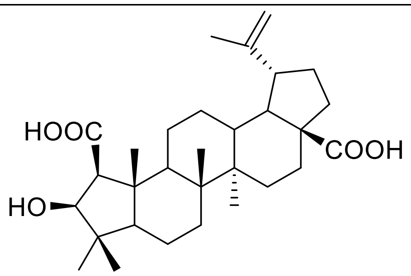

epiceanothic acid (**2**)

References

1. Voutquenne-Nazabadioko L, Gevrenova R, Borie N, Harakat D, Sayagh C, Weng A, *et al.* 2013. Triterpenoid saponins from the roots of *Gypsophila trichotoma* Wender. *Phytochemistry*. **90:** 114-127.
2. Vinh LB, Jang H-J, Phong NV, Cho K, Park SS, Kang JS, *et al.* 2019. Isolation, structural elucidation, and insights into the anti-inflammatory effects of triterpene saponins from the leaves of *Stauntonia hexaphylla*. *Bioog Med Chem Lett.* **29:** 965-969.
3. Vinh LB, Lee Y, Han YK, Kang JS, Park JU, Kim YR, *et al.* 2017. Two new dammarane-type triterpene saponins from Korean red ginseng and their anti-inflammatory effects. *Bioorg Med Chem Lett.* **27:** 5149-5153.
4. Duyen NT, Vinh LB, Phong NV, Khoi NM, Long PQ, Hien TT, *et al*. 2022. Steroid glycosides isolated from *Paris polyphylla* var. *chinensis* aerial parts and paris saponin II induces G1/S-phase MCF-7 cell cycle arrest. *Carbohydr Res.* **519:** 108613.
